# Do People Know Adequately about Leptospirosis? A Knowledge Assessment Survey in Post-outbreak Situation in Sri Lanka

**Published:** 2010

**Authors:** Suneth B Agampodi, Thilini C Agampodi, Eranga Thalagala, Sahan Perera, Shashika Chandraratne, Shantushya Fernando

**Affiliations:** 1 Department of Community Medicine, Faculty of Medicine and Allied Sciences, Rajarata University of Sri Lanka, Saliyapura, Sri Lanka

**Keywords:** Leptospirosis, Knowledge, Sri Lanka

## Abstract

**Objectives:**

Sri Lanka experienced the worst ever outbreak of leptospirosis in 2008. One major determinant of control and prevention of communicable diseases is public awareness on the disease. The purpose of the present study was to determine the awareness on leptospirosis among public Sri Lanka.

**Methods:**

A national household survey was carried out as a part of research methodology training of first year medical undergraduates in Rajarata Medical School. Each student visited 10 households surrounding his/her house to complete the interviewer administered questionnaire. The questionnaire was based on the factsheet published by the Epidemiology Unit for public health.

**Results:**

Altogether 602 participants from 14 districts participated in the study. Of them 93.7% were aware of rat as a reservoir animal, but only 3% were aware of the role of cattle and buffalo. Contact with infected water as a mode of transmission was reported by 57.9% of the population. Only 30.8% of the subjects were aware of that the infection can go through skin breeches. Farming as a risk activity was reported by 63.5% of the patients, but knowledge on other exposure activities were less than 20%. Paddy field work and cleaning garbage were correctly identified as risk occupations by 89.7% and 27.6% of the sample, respectively. Respondents were aware of fever (86%), malaise (30.8%), headache (29.6%) and muscle tenderness (28.8%) as main clinical features of the disease. Most of them (73.7%) knew leptospirosis as a lethal condition and 39.5% were aware of chemoprophylaxis.

**Conclusions:**

Although there is not adequate information on MDD prevalence in some areas of Iran, the overall current prevalence of MDD in the country is high and females are at the greater risk of disease.

## INTRODUCTION

Leptospirosis is an emerging infectious disease with worldwide distribution. The disease is endemic in humid, tropical, and subtropical areas of the world where most of the developing countries are located.^1^ In Asia Pacific region, Latin America and in Southeast Asia, it is highly prevalent and there has been a marked increase in the number of outbreaks and cases reported during the last two decades.^2^ Even though the disease is mostly endemic in rural settings, an increasing number of cases and frequent outbreaks among urban dwellers is a recent finding worldwide.^3^–^5^ In Sri Lanka, leptospirosis is an endemic disease and frequent outbreaks were observed once every 3-4 years, during the last two decades. In 2008, Sri Lanka reported the worst ever outbreak of leptospirosis in its history with more than 7000 reported cases. The disease incidence during this year was 35.7/100,000 population and the case fatality rate was 2.9%.^6^ With the wake of 2008 massive outbreak of leptospirosis, the epidemiology unit of Sri Lanka and the ministry of health launched a programme on leptospirosis control, several consultative meetings were conducted and the fact sheets and guidelines were prepared. These plans included implementing surveillance, creating awareness, and improving clinical management and chemoprophylaxis.^7^ Epidemiological fact sheets on leptospirosis and awareness programs on disease prevention were already in place through the public health system in leptospirosis endemic districts, which were strengthened after this outbreak. In prevention of leptospirosis, awareness on disease existence, knowledge and health behaviours play a key role. Even though knowledge itself is not adequate in changing behaviours, knowledge and awareness make the platform for behaviour change. It is important to study whether the public awareness programs are effective in improving the knowledge of people regarding this important disease in Sri Lanka. Formal assessment of awareness and knowledge of leptospirosis among general public has not been carried out in Sri Lanka previously. Purpose of the present paper is to discuss the finding of an islandwide survey on leptospirosis knowledge among general public.

## METHODS

The data discussed in the present paper was collected during a module on “Introduction to medical research” to new recruit medical undergraduates of Rajarata University of Sri Lanka. As part of introduction to questionnaire surveys for the 2008-9 batch of medical students, a data collection field training exercise was carried out in December 2009, using a questionnaire developed by the Investigators. The questionnaire used was an interviewer administered, structured questionnaire, based on the epidemiological fact sheet published by the epidemiology unit. Questionnaire was design to assess the awareness of disease prevalence in residing area, knowledge on reservoirs, mode of disease transmission, probable occupational exposures, clinical features, complications and disease prevention. All questions included to assess knowledge were multiple response questions, where all given answers were correct. Each medical student visited neighbours of his/her residence to complete the questionnaire. As a part of this exercise, students were given factsheets to disseminate knowledge on leptospirosis after the interview. Children less than 10 years of age were excluded from the study sample. Each participating medical student’s home residence was considered as a central point for cluster sampling and the size of the cluster was 10. A basic training on data collection procedure, probing and using questionnaire was given to the data collectors prior to data collection.

## RESULTS

**Figure 1:**
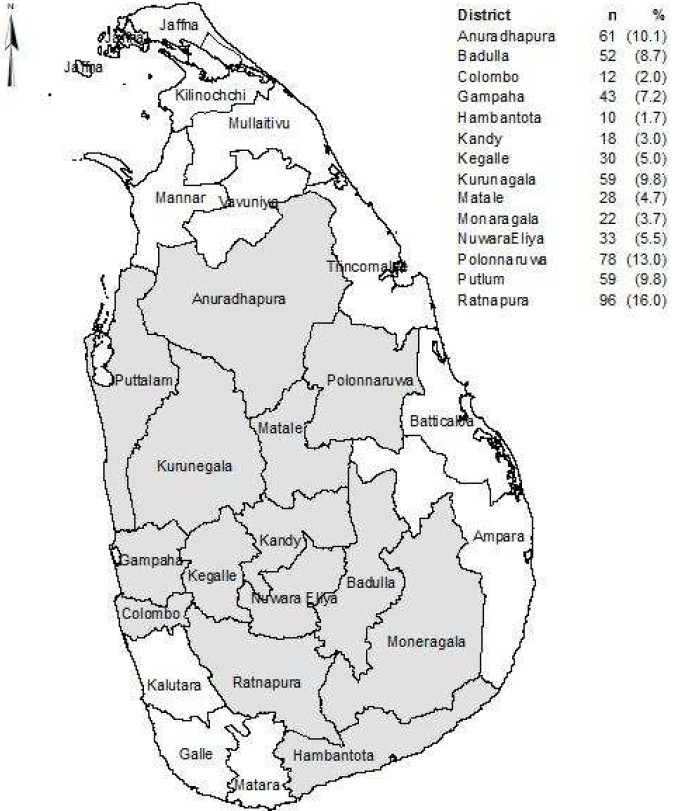
Districts selected for the survey and the number of participants selected from each district.

A total of 601 participants responded to the survey from 63 clusters, from 13 districts representing seven out of nine provinces in Sri Lanka. Figure 1 shows the selected districts and the number of participants responded to the survey in each district. Table 1 shows the demographic profile of the study sample.

**Table 1:** Characteristics of the study sample.

Socio-demographic characteristics	(n=601)	
	n	(%)
**Sex**		
Female	306	(50.9%)
Male	269	(44.8%)
Data missing	26	(4.3%)
**Educational attainment**		
No formal education	13	(2.2%)
Primary	18	(3.0%)
Post-primary	226	(37.6%)
Secondary	264	(43.9%)
Tertiary	80	(13.6%)
Data missing	13	(2.2%)
**Occupation**		
Professionals	17	(2.8%)
Associate professionals	120	(19.9%)
Clerical and related fields	109	(18.1%)
Skilled manual workers	108	(17.9%)
Unskilled manual workers	38	(6.3%)
Housewives	149	(24.8%)
Students	28	(4.7%)
Unemployed/data missing	33	(5.5%)

Mean age of the sample was 40.9 years (SD 13.0). Sample showed a normal distribution of age. One third of the respondents were school/university students and housewives. Of the employed people, majority were in social class 3-5.

Awareness on the disease prevalence was evaluated by asking “How many cases of leptospirosis (rat fever) have been reported from your area recently? Answers to the particular question are presented in Table 2 according to the endemicity of the area.

**Table 2:** Awareness on disease prevalence among the study sample.

	Endemic areas		Non-endemic areas	
	n	%	n	%
No idea	101	(35.3%)	105	(33.7%)
Never heard of leptospirosis in the area	70	(24.5%)	100	(32.1%)
Very few cases	75	(26.2%)	70	(22.4%)
Several cases	31	(10.8%)	31	(9.9%)
Large number of cases	9	(3.1%)	6	(1.9%)
Total	286	(100%)	312	(100%)

Awareness of reported cases was not different between endemic and non-endemic districts. More than one third of the sample reported that they never heard of leptospirosis cases from their areas. Table 3 shows the knowledge of respondents on probable reservoirs, disease transmission, occupational exposures, clinical features, complications and prevention of disease.

**Table 3:** Knowledge on leptospirosis among the study samples.

	(n=601)	

Knowledge on leptospirosis	n	%
**What are the animals that can harbour “rat fever ” infection?**		
Rats	561	(93.7%)
Other rodents	132	(22.0%)
Cattle	9	(1.5%)
Buffaloes	10	(1.7%)
Dogs	0	(0.0%)
**What are the ways that you can get infections from those animals?**		
Direct contact with infected urine	285	(47.7%)
Drinking contaminated water	152	(25.4%)
Contact with contaminated water	346	(57.9%)
Contact with contaminated wet soil	141	(23.6%)
**What are the at risk occupation to get “rat fever ”?**		
Paddy field work	534	(89.7%)
Mine work	64	(10.8%)
Cleaning garbage/ drainage	164	(27.6%)
Animal husbandry	80	(13.4%)
**What are the common symptoms of “rat fever ”?**		
Fever	511	(86.0%)
Malaise	183	(30.8%)
Headache	176	(29.6%)
Muscle tenderness	171	(28.8%)
Oliguria	88	(14.8%)
Jaundice	87	(14.6%)
Red eyes	119	(20.0%)
**What could happen as complications of “rat fever ”?**		
Renal problems	233	(39.5%)
Haemorrhages	51	(8.6%)
Heart failure	90	(15.3%)
Death	435	(73.7%)
**What are the methods that could be useful in preventing “rat fever ”?**		
Use of chemoprophylaxis before paddy field work	296	(50.3%)
Wearing boots and gloves	317	(53.9%)
Drinking boiled cool water	118	(20.1%)
Proper waste disposal	154	(26.2%)
Avoiding flood water	119	(20.2%)

^*^All responses provided were correct and the percentages were calculated separately for each response.

Of the total sample studied, 69 reported their main occupation as paddy field farming. Knowledge of farmers was compared with the rest of study sample to assess the knowledge of high risk group. Of the 29 knowledge questions assessed, in only three questions farmers showed higher levels of knowledge. Malaise was known by 28/69 (40.6%) farmers compared to 154/532 (28.9) other occupational groups (chi-square 3.915, P=0.048) as a symptom of leptospirosis; 27.5% (n-19) of farmers were aware of jaundice as a main feature of leptospirosis compared to 12.8% (n=68) of others (chi-square 10.74, P=0.001). Among farmers, 63.8% (n=44) knew that there is a drug that they can use as a prophylactic measure whereas only 47.2% of others were aware of chemoprophylaxis (chi-square 6.725, P=0.01). Participants from endemic areas (as defined by the Epidemiology Unit of Sri Lanka) also had significantly higher knowledge on chemoprophylaxis compared to participants from non-endemic districts (64.0% and 35.6%, respectively, chi-square 48.4, P<0.001). However, no knowledge difference was observed with regards to other areas assessed between participants from endemic and non-endemic areas. Level of knowledge was not significantly associated with age, sex and educational level of the participants.

## DISCUSSION

The main objective of the present study was to assess the knowledge and awareness on leptospirosis in general public. The study sample was drawn from seven provinces excluding north and east because the questionnaire was developed only in Sinhalese. Sample was not representing the actual population residing in these districts due to the procedure followed for sampling. However, the selected sample was representing a wide range of occupational categories from rural and urban areas both from leptospirosis endemic and non-endemic districts. Knowledge assessment was based on the information of the epidemiological fact sheet; therefore, some aspects of knowledge are not included in the questionnaire. Awareness on disease prevalence in one’s own community was low among most of the people who live in endemic areas where there were several hundred reported cases during the 2008 outbreak of leptospirosis. Lack of awareness leads to reduce the perceived threat of the disease, which could minimize taking preventive measures. Knowledge on the reservoirs was mainly restricted to rat and other rodents. This finding was not surprising with the existing knowledge even among health care professionals. Leptospirosis is traditionally known as “rat fever” for a long time in Sri Lanka. Studies on leptospirosis in Sri Lanka from 1959 to 2003 showed that most of the authors were preoccupied with the idea of rat as the only source of infection.^8^–^10^ In these studies, some of the authors discussed the presence of rats at home and or in working places, but not other animals. It was evident that authors have not tried to group the available evidence to see prevalent serovars and cross compare those with natural maintenance hosts. This issue was first raised by Agampodi et al. in 2008.^11^ At present, experts are well-aware of this fact, however the public is unaware of this as shown in the present study.

Most of the respondents were not aware of the main mode of transmission, which is through contaminated water. Only 34% knew about this mode while 47% of the sample knew that the direct contact with urine from infected animal could cause infection. On the other hand, paddy field work was the well-known exposure for contracting leptospirosis, whereas other activities were known by less than 7% of the respondents. One of the most alarming features was the lack of awareness on clinical features which could delay hospital admissions thus end up with more complications. Nevertheless, around 50% of respondents knew that complicated cases could die and 40% knew about renal complications. Other than the knowledge on chemoprophylaxis, participants from endemic and non-endemic areas had the same level of knowledge. The use of doxycycline chemoprophylaxis, which showed to be ineffective in preventing leptospirosis, was known to 51% of all participants. Significantly higher percentage of farmers knew about the chemoprophylaxis.^12^

## CONCLUSION

Despite the recent massive outbreaks of leptospirosis and control programs launched by the epidemiology unit, public knowledge on leptospirosis is poor in Sri Lanka. Divisional level and grassroots level public health workers should put more effort in awareness raising and health education programs in order to achieve good control and prevention of leptospirosis in Sri Lanka.
